# Treatment-seeking for selected reproductive health problems: behaviours of unmarried female adolescents in two low-performing areas of Bangladesh

**DOI:** 10.1186/1742-4755-11-54

**Published:** 2014-07-17

**Authors:** Humayun Kabir, Nirod Chandra Saha, Andrea L Wirtz, Rukhsana Gazi

**Affiliations:** 1 International Centre for Diarrhoeal Disease Research, Bangladesh (icddr,b), Mohakhali, Dhaka 1212, Bangladesh; 2 Department of Emergency Medicine, Johns Hopkins Medical Institute, Baltimore, Maryland, USA

**Keywords:** Female adolescents, Menstrual problem, STIs, Treatment-seeking, Healthcare facilities, Urban, Rural, Bangladesh

## Abstract

**Purpose:**

The reproductive health needs of unmarried adolescents in Bangladesh are largely unmet. This study aimed to explore treatment-seeking behaviour of unmarried female adolescents for selected reproductive health (RH) concerns in two low-performing areas of Bangladesh.

**Methods:**

As part of a large community based-project, a cross-sectional survey was conducted from November 2006 to March 2007. From each of two select study areas, 800 unmarried female adolescents aged 12–19 years were selected for participation by simple random sampling through household listing and were recruited into the study. Trained interviewers administered a structured questionnaire to participating female adolescents. Descriptive and bivariate analytic methods were used compare RH conditions and healthcare seeking behaviour of adolescents across urban and rural settings.

**Results:**

Approximately 50% of the sample reported experiencing menstrual problems in the last year. The predominant problems reported by participants included: lower abdominal pain, back pain, irregular menstruation, and excessive bleeding during menstruation. Irrespective of study area, only 40% of the female adolescents with menstrual problems sought treatment from qualified physicians. Otherwise, utilization of healthcare facilities and care providers for reported problems varied significantly by rural and urban areas. Higher proportions of adolescents in the urban setting (15%) also reported recent symptoms of sexually transmitted infections (STIs), compared to those in the rural setting (9%; p < 0.001). Across sites, however, self-treatment was the most commonly reported method of care for those who experienced any symptoms of STI.

**Conclusions:**

In general, treatment-seeking behaviours by unmarried female adolescents was low for menstrual problems. A vast majority of unmarried female adolescents practiced self-care for symptoms of STIs while only small proportions sought treatment from qualified physicians. These findings emphasize the need for offering relevant information on RH issues and introducing confidential adolescent-friendly reproductive healthcare facilities to enable unmarried female adolescents access to RH services when necessary.

## Introduction

Adolescents aged 10–19 years constitute the largest-growing segment of the world population. Globally, one in every 5 is an adolescent [1]. In south Asian countries, current healthcare systems scarcely address the health needs of adolescents. Furthermore, they are inadequately informed about the symptoms and consequences of reproductive health conditions [2]. Although a large number of adolescents suffer from reproductive health problems, a vast majority of them do not seek healthcare for these conditions [3].

Although Bangladesh has achieved some progress towards several targets of the Millennium Development Goals (MDGs) [4], but the birth rate among adolescents is still high, 118 per 1,000 women (MDG 5) [5]. According to the 2011 National Demographic Surveillance Report, about 25% of married adolescents had given birth by the age of 19 [5]. Although the current prevalence of HIV among the general population in Bangladesh is still below an epidemic level (MDG 6) [6], adolescents and youths are often considered to be vulnerable to acquiring HIV infection due to higher risk sexual behaviour for [7,8]. As estimated by the national program on HIV in Bangladesh, only 17.7% of adolescents have comprehensive knowledge of HIV transmission and prevention methods [9].

The heterogeneity of the healthcare system infrastructure across rural and urban settings of Bangladesh must also be considered when addressing RH problems for adolescents. National-level community-based surveys in Bangladesh revealed that there are variations in choices and utilization of specific types of health-care services by population groups [10]. Moore *et al*. (2006) found that youth healthcare-seeking from existing healthcare facilities for RH conditions was considerably lower compared to healthcare-seeking for other general health problems in Bangladesh [11].

Bangladesh’s Strategic Plan for Health, Population and Nutrition Sector Development Programme 2011–2016, has prioritized safe motherhood, family planning, menstrual regulation, and care for post-abortion complications and management of sexually transmitted infections (STI), with specific guidelines laid out in the National Reproductive Health Strategy [6]. Both Directorate General of Health Services and Directorate General of Family Planning implement reproductive health services through their programs on maternal, neo-natal, and child health (MNCH), reproductive and sexual health, including family planning [6]. Married women are the main beneficiaries for these services. In Bangladesh, however, unmarried female adolescents receive limited attention for healthcare issues by these existing programs.

From 2005–2008, the United Nations Population Fund (UNFPA), in collaboration with the National Institute of Population Research and Training (NIPORT), and icddr,b implemented the “Demand-based Reproductive Health Commodity Project (DBRHCP).” This intervention focused on training of government service providers, disseminating behaviour change materials within the targeted communities, and employing community-based health promoters to foster linkages between the community and providers. To understand the situation for the population of unmarried female adolescents, we explored the healthcare-seeking behaviour of these adolescents for selected reproductive health problems in one rural area and one urban slum area of Bangladesh to develop interventions under DBRHCP.

## Materials and methods

### Study design and population

A cross-sectional survey was conducted among 1,600 unmarried female adolescents aged 12–19 years during November 2006 to March 2007 under DBRHCP. Among the four wards of Dhaka City and two rural sub-districts where the DBRHCP was implemented, one rural and one urban slum were selected for this study. Selection was based on consideration of current RH indicators and DBRHCP selected Nabiganj Upazila (sub-district) in the Sylhet division, and one urban slum of Dhaka city in the Dhaka division of Bangladesh. Sylhet division is known as a low-performing area with the lowest RH indicators: the contraceptive prevalence rate (CPR) is low; maternal and infant mortality rates and total fertility rates (TFR) are high; and healthcare-seeking behaviour is low compared to the national level indicators [12]. The use of health facilities also varies widely between urban and rural areas [13].

The sample size was estimated based on a 95% confidence with 90% power and a 25% non-response rate. The sample size calculation was based on prior research conducted by icddr,b in Abhoynagar and Mirsarai, which estimated that approximately 5% of adolescents from the rural area utilized services from government facilities or NGOs [14]. This gave an effective target sample size of 800 participants per site. All households in the target areas were enumerated to collect basic information regarding age, sex, marital status, socio-economic condition, and other similar variable to develop the sampling frame under DBRHCP. According to this enumeration, there were 54,116 households in Nabiganj and 29,904 in the Dhaka urban slum. We selected 800 households from the sampling frame through a simple random sampling process.

A total of 1,600 unmarried female adolescents were selected from the two study areas. Inclusion criteria required participants to be female, age 12–19 years and currently unmarried. We excluded respondents if more than one adolescent fulfilled the criteria from the same household. Adolescents who were not residents in the area were also excluded. In households where potential participants resided but were absent on the first attempted visit, up to three subsequent attempts were made to reach absentees.

### Survey measures and data collection

Experienced female interviewers who had previously worked in different public health research studies were recruited to serve as interviewers for this study. Selected interviewers received one month extensive training on adolescent health, reproductive health services, menstrual problems, HIV and STIs, existing health service facilities and availability of services, and interview techniques. Trained interviewers interviewed unmarried female adolescents using structured questionnaire. Each day after returning from the field, the interviewers crosschecked the completed questionnaires. The field supervisors reviewed each of the questionnaires and conducted regular spot-checking to maintain data quality. An experienced field research manager coordinated the overall field activities.

Prior to survey implementation, the study questionnaire was field tested and followed with a debriefing session to identify any errors or necessary corrections to the questionnaire. The questionnaire included items to assess knowledge and perceptions of menstrual or STI-related conditions, individual experiences of menstrual or STI-related conditions, and treatment-seeking behaviours. Menstrual conditions included: lower abdominal pain, back pain, excessive bleeding, irregular menstruation patterns, and others, such as sore in inner side of thigh, nausea, vertigo, headache, weakness, and the like. STI-related items focused on knowledge of STIs and STI symptoms, experiences of STI or STI-related symptoms, and whether treatment was sought for such suspected STI symptoms. STI-related symptoms included burning during urination, genital ulcer/sore, and excessive bleeding. These questions were prompted using the following two questions: “Have you heard about sexually transmitted diseases, what are those?” and “Have you experienced any of the following STI symptoms in the last one year?”. Recall periods for menstrual or STI conditions and treatment-seeking behaviours focused on experiences during the past year (12 months). General health and healthcare seeking behaviours for illness was assessed and used a recall period of the last three months.

### Ethical consideration

Ethical approval for the study was obtained from the Ethical Review Committee (ERC) of icddr,b. The parents of unmarried female adolescents were asked for consent to allow their daughters to participate in the study. Once parental consent was obtained, the study team then approached the respondents. The parental non-response rate was 7% in rural area and 10% in urban area, but had already been corrected for in overall sample size estimation. During the informed consent with select adolescents, none of the potential participants refused to participate. Data collection was anonymous and privacy was maintained during data collection. All interviews were conducted interview in a private corner/place of the household. To ensure confidentiality and anonymity, only identification numbers were used during data collection.

### Data analysis

Descriptive analysis was conducted to estimate distributions of relevant characteristics of the sampled populations. A comparative analysis of urban and rural areas was conducted Bivariate analysis was conducted to compare urban and rural sites with respect to participant age, education, menstrual conditions, treatment sought for menstrual problems, reported STI symptoms, treatment sought for STI symptoms, and the use of health facilities for any general health illness. Proportions, 95% confidence intervals (CIs), and p values were calculated for select variables. SPSS version 10.0 (IBM Corp, Armonk, NY) was used for all statistical analysis.

## Results

Table 1 displays the demographic characteristics of participants across the two sites. Participants from urban Dhaka were significantly younger compared to rural Nabiganj, with 44% aged 12–14 years compared to 27%, respectively (p < .001). On the other hand, the proportion of participants in the age-groups of 15–17 and 18–19 years were significantly higher in rural Nabiganj (50%; and 23%; p < .01) compared to urban Dhaka (39%, p < .001 and 17%) respectively. In both areas, 90% of adolescents had ever been enrolled in school. Of those, the proportion of adolescents who completed above the 5^th^ grade was significantly higher in Nabiganj (53%) compared to the Dhaka slum (43%; p < 0.001).

**Table 1:** Profile of unmarried female adolescents across urban and rural study areas of Bangladesh, 2006–2007

	**Urban**	**Rural**	
	**Dhaka (n = 800)**	**Nabiganj (n = 820)**	
**Age in years**	% (95% CI)	% (95% CI)	**P-value**
12-14 years	44% (40.6-47.4)	27% (24.0-30.0)	***
15-17 years	39% (35.6-42.4)	50% (46.6-53.4)	***
18-19 years	17% (14.4-19.6)	23% (20.1-25.9)	**
**Ever enrolled in school**	90% (87.8-92.2)	90% (87.8-92.2)	NS
**Educational attainment**	(n = 720)	(n = 738)	
1-5 years	57% (53.4-60.6)	47% (43.4-50.6)	***
≥ 6 years	43% (39.4-46.6)	53% (49.4-56.6)	***

*p < 0.05; **p < 0.01; ***p < 0.001.

Table 2 displays the self-reported menstrual conditions and treatment seeking behaviours of participants across the two sites. Almost similar proportions of adolescents reported experiencing any menstrual problem during the last year in urban Dhaka (50%) and in rural Nabiganj (47%) areas. Among them, more than half of the adolescents reported back pain as the most common menstrual condition and less than one-tenth of adolescents reported irregular menstruation across the two sites. A significantly higher proportion of adolescents in rural Nabiganj (28%) reported lower abdominal pain compared to urban Dhaka (22%; p < 0.05). Self-reported excessive bleeding was highly significant in rural Nabiganj (13%) compared to urban Dhaka (6%; p < 0.001). Nausea, headache, vertigo and weakness were also significantly higher among female adolescents in rural Nabiganj (43%) compared to urban Dhaka (32%; p < 0.01).

**Table 2:** Experiences, types, treatment-seeking for menstrual problems by unmarried female adolescents in urban and rural sites in Bangladesh

	**Urban**	**Rural**	
	**Dhaka (n = 778)**	**Nabiganj (n = 820)**	
**Characteristic**	**% (95% CI)**	**% (95% CI)**	**P-value**
**Experiences of menstrual- problems (last 12 months)**	50% (46.5-53.5)	47% (43.6-50.4)	
**§Types of menstrual problems experienced**	(n = 390)	(n = 382)	
Lower abdominal pain	22% (17.9-26.1)	28% (23.5-32.5)	*
Pain in the back	52% (47.0-57.0)	54% (49.0-59.0)	
Excessive bleeding	6% (3.6-8.4)	13% (9.6-16.4)	***
Irregular menstruation	6% (3.4-8.4)	9% (6.1-11.9)	
Nausea, headache, vertigo and weakness	32% (27.4-36.6)	43% (38.0-48.0)	**
**Proportion of adolescents who received treatment**	13% (9.7-16.3)	27% (22.5-31.5)	***
**§Types of healthcare providers consulted for treatment of menstrual problems**	(n = 52)	(n = 102)	
Qualified physician	40% (26.7-53.3)	39% (29.5-48.5)	
Nurse/Family Welfare Visitor/Health Worker/Family Welfare Assistant	6% (-0.5-12.5)	2% (-0.7-4.7)	
Indigenous practitioners	17% (6.8-27.2)	33% (23.9-42.1)	*
Village doctor	19% (8.3-29.7)	19% (11.4-26.6)	
Pharmacy	17% (6.8-27.2)	7% (2.0-12.0)	*

§Multiple responses; *p < 0.05; **p < 0.01; ***p < 0.001.

The proportion of the adolescents who have reported seeking treatment for any menstrual problems was significantly higher in rural Nabiganj (27%) compared to urban Dhaka (13%; p < 0.001). Among those seeking care, approximately 40% of adolescents sought treatment from qualified physician (a healthcare provider who has obtained formal medical degree) across both areas. In the rural area, a higher proportion of adolescents (33%) consulted indigenous practitioners for menstrual problems compared to the urban area (17%; p < 0.05). In contrast, a higher proportion of the adolescents in the urban area (17%) received treatment from pharmacies compared to rural area (7%; p < 0.05).

Table 3 displays STI symptoms and treatment seeking behaviour across the two study sites. Self- reported STI symptoms were heterogeneous across the study areas. A significantly higher proportion (15%) of adolescents in urban Dhaka reported STI symptoms during the last year compared to rural Nabiganj (9%; p < 0.001). Reported STI symptoms were similar across the sites and included: burning during urination, genital ulcer/sore, and excessive bleeding. A higher, but modest, proportion of adolescents in Nabiaganj (16%) sought treatment from a qualified physician compared to Dhaka (6%; p < 0.05). A similar trend was observed with seeking treatment from indigenous practitioners, as higher, but non-significant, 6% of participants in Nabiganj sought treatment from indigenous practitioners compared to 3% in urban Dhaka. Self-treatment was the most commonly reported care for STI symptoms in both study areas.

**Table 3:** Reported STI symptoms, and health care providers consulted by unmarried female adolescents for STI treatment in the last year, in urban and rural sites in Bangladesh

	**Urban**	**Rural**	
	**Dhaka (n = 800)**	**Nabiganj (n = 795)**	
**RH Characteristic**	% (95% CI)	% (95% CI)	**P-value**
**Reported STI symptoms in the last year**	15% (12.5-17.5)	9% (7.0-11.0)	***
**Reported STI signs/symptoms**	**(n = 124)**	**(n = 68)**	
Burning during urination	8% (3.2-12.8)	4% (-0.7-8.7)	
Genital ulcer/sore	4% (0.6-7.4)	2% (-1.3-5.3)	
Excessive bleeding	8% (3.2-12.8)	5% (-0.2-10.2)	
**§Types of healthcare providers consulted for STI treatment**	**(n = 124)**	**(n = 68)**	
Qualified physician	6% (1.8-10.2)	16% (7.3-24.7)	*
Self-treatment	80% (73.0-87.0)	68% (56.9-79.1)	
Indigenous practitioners	3% (0.0-6.0)	6% (0.4-11.6)	
Pharmacy	4% (0.6-7.4)	2% (-1.3-5.3)	

§Multiple responses; *p < 0.05; ***p < 0.001.

Table 4 displays participant utilization of health facilities for general health concerns across the two sites. Higher proportions of the adolescents in rural Nabiganj (14%) visited any health facility in the last three months for any general health problem, compared to those in urban Dhaka (7%; p < 0.001). Among them, significantly higher proportions of adolescents received treatment from the public-sector facilities in rural Nabiganj (50%) compared to urban Dhaka (8%; p < 0.001). Similarly, in urban Dhaka, significantly higher proportions of adolescents utilized services provided by non-governmental organizations (NGOs) (26%; p < 0.01) visited private healthcare facilities (62%; p < 0.05)

**Table 4:** Utilization of health facilities by unmarried female adolescents for any general health problems in urban and rural sites in Bangladesh

	**Urban**	**Rural**	
	**Dhaka n = 766**	**Nabiganj n = 611**	
**Health characteristic**	% (95% CI)	% (95% CI)	**P-value**
**Proportion that visited any health facilities in the last three months**	7% (5.2-8.8)	14% (11.2-16.8)	***
**§Types of health facility visited by adolescents in the last three months**	(n = 53)	(n = 88)	
Public sector	8% (0.7-15.3)	50% (39.6-12.3)	***
NGOs	26% (14.2-37.8)	7% (1.7-12.3)	**
Private sector	62% (48.9-75.1)	42% (31.7-52.3)	*

§Multiple responses; *p < 0.05; **p < 0.01; ***p < 0.001.

## Discussion and conclusions

Findings from this study demonstrate the reproductive health needs of rural and urban residing unmarried adolescents. Despite the prevalence of recent menstruation related conditions and STI symptoms, relatively low proportions of adolescents seek treatment for these concerns. In particular, treatment seeking by adolescents from qualified physician in both areas was poor for any menstrual problems. Likewise, self-treatment for STI symptoms was the most common source of care in across rural and urban study areas. Overall, there was significant variation across rural and urban settings with respect to the types of facilities utilized for reported reproductive health problems.

Treatment-seeking behaviours by adolescents for menstrual conditions and STI may be explained by individual levels of comfort and familiarity with providers as well as with accessibility of the services. The increased use of indigenous practitioners and pharmacies for such problems may be due to their easy accessibility or privacy of services. In rural areas, adolescents may have preferred receiving treatment from homeopathic practitioners or *kabiraj,* possibly because they were more accessible, acceptable and affordable to them. For similar reasons, adolescents in the urban area may have chosen to visit pharmacies to receive treatment for any menstrual problems. Given the potential stigmas related to seeking care for menstrual conditions or STI, unmarried adolescents may prefer locations that offer greater discretion to those that may be well-qualified. Future studies may benefit by exploring the reasons for selecting/preferring particular healthcare providers or facilities by adolescents.

The present study found that self-treatment was the most common form of care for STI symptoms. General shyness, fear of stigma, or fear of breached confidentiality may explain the low proportion of adolescents who seek care from qualified providers. Other research from Bangladesh reveal that self-care/self-treatment was the most common choice of care among adult women of all ages for general illness, followed by use of a qualified physician and then by use of indigenous practitioners [15]. Thus, such discretion may be often a cultural norm in Bangladesh and preference for self-treatment may also be due to the lack of presence of female health professionals at the facilities. However, more research is needed in this specific area to confirm these hypotheses. Data from India highlight the differences in seeking care between married and unmarried women. In India, some 57% of married and 66% of unmarried women experiencing STI symptoms reported failing to seek treatment. Among married women who did seek treatment, two-fifths received treatment from formal medical care providers (11% from the public sector and 28% from the private sector) compared to the one-third of the unmarried women who obtained formal treatment (10% and 20% respectively). Only 3-4% of each group trusted traditional healthcare providers or home remedies. These disparities in healthcare-seeking behaviors highlight the barriers that unmarried women face when seeking care for STI, likely attributable to stigma of having an STI out of wedlock, and the lack of capacity among the public-sector facilities to serve unmarried youth [16].

This study also revealed that less than one-tenth of the adolescents in urban areas visited public health facilities during the last three months for any general health problems. These findings are consistent with national community-based surveys in Bangladesh, which estimate that one-tenth of households report at least one member receives treatment from public healthcare facilities [10]. Among rural adolescents seeking healthcare for general health problems in this study, the most common source of healthcare was from the public sector. In urban settings, NGOs and private healthcare facilities were the most commonly utilized healthcare facilities. Variations in utilization of healthcare facilities may be associated with general distribution and availability of health services in Bangladesh. Generally, the public sector provides the main source for healthcare services in rural areas, while NGOs and the private facilities are the most commonly available and utilized facilities in urban areas.

Taken together, research on healthcare utilization among unmarried adolescents suggests that innovative and confidential interventions are needed to address RH concerns among this population. Such an intervention may build on the promising findings of the Mobile Alliance for Maternal Action (MAMA) programme that was implemented in 2012 in Bangladesh, as well as in South Africa and Indonesia. This programme aimed to provide care for pregnant women, new mothers, and their families during pregnancy and delivery. Under this initiative, 20% of women with low incomes or in poverty in the select sites served were beneficiaries and received free use of mobile phones under the MAMA program. Mobile voices messages and text messages provided a range of educational information relating to pregnancy and care, as well as reminders to attend medical check-ups. The MAMA program demonstrates the use of information communication technology (ICT) interventions in Bangladesh, which could be adapted and applied to address the reproductive health needs of adolescents [17]. Approximately nine in 10 households in urban areas and more than seven in 10 households in rural areas of Bangladesh are mobile phone users [5], and adolescents constitute a large proportions of the total users. Considering the sensitive nature of these RH issues, private and innovative approaches based on ICT, particularly using mobile phones, could be pilot-tested to address reproductive health concerns among unmarried adolescents. Introduction of ICT related healthcare interventions at the school-level may also serve to improve the RH aspects of unmarried adolescents.

Reviews of school-based healthcare services have demonstrated evidence of acceptability among adolescents; cost-benefit with respect to adolescent health; reduction of health disparities, and improvements in health-seeking behaviors. Unfortunately, the majority of researches on this topic come from North America [18]. Research from a more similar setting in India has also demonstrated promising alternative methods to address low levels of health-care-seeking behaviour for reproductive conditions and STI [3,19]. In recognition of adolescents as an underserved, vulnerable group who are in need of information on reproductive health and related services, the National Population Policy of India introduced a school-based adolescent friendly intervention in urban Mumbai. The intervention was subsequently scaled up across different parts of India. Services operated two days per week for two hours per day and were managed by a medical doctor and two counsellors*.* Adolescents decided the convenient times during which to hold services, such as after school hours and weekend*s.* Services included the provision of information, counselling, and medical consultations free of charge. Findings from the intervention demonstrated that 43% of enrolled girls and 35% of enrolled boys visited the centre on their own during the one-year project period. Among adolescents experiencing RH problems, however, only one-fifth reported voluntarily to the centre [20].

Findings from this study should be viewed in light of several limitations. Data were obtained from only one rural area of one division and one urban area of another division out of seven divisions and the generalizability of these findings to the wider population in Bangladesh or the region is unclear. Additionally, only quantitative analysis was conducted for this study. Use of both quantitative and qualitative methods would have enriched understanding of the context of RH issues and treatment-seeking behaviours of unmarried adolescents.

## Conclusion

The National Reproductive Health Strategy of Bangladesh prioritizes on safe motherhood, family planning, menstrual regulation, care for post-abortion complications, and management of STI. While both the Directorate General of Health Services and Directorate General of Family Planning implement reproductive health services through their programs on maternal and child health and reproductive, only married women truly benefit from these services [6]. Overall, the study presented here demonstrates that treatment-seeking by unmarried female adolescents was low for menstrual and STI-related concerns, while the vast majority of the unmarried female adolescents opted for self-care for STI symptoms. These findings emphasize the need for improved accessibility to relevant information on RH issues. Existing health facilities should be made more adolescent-friendly to improve the health status of the unmarried female adolescents.

Future research would benefit from exploring the reasons why unmarried adolescents prefer self-treatment, why they do not seek care from qualified professionals, and what characteristics would be ideal in adolescent-friendly facilities. Taken together, these findings may assist policy-makers’ efforts in improving existing health systems by establishing adolescent-friendly healthcare services across urban and rural settings, ultimately aiming to improve the reproductive health status of female adolescents in Bangladesh.

## Competing interests

The authors declare that they have no competing interests.

## Authors’ contribution

HK drafted the manuscript and was involved in field implementation and data analysis of the study. NC did statistical analysis. AW revised the manuscript and provided technical support to finalize the manuscript. RG was involved in designing of the study and provided overall guidance to prepare the manuscript & acted as a mentor. All authors read and approved the final version of the manuscript.
